# Stability of Important Veterinary Antibiotics Amoxicillin, Sulfadiazine, and Trimethoprim in Practice-Relevant Model Solutions

**DOI:** 10.3390/antibiotics12020214

**Published:** 2023-01-19

**Authors:** Friederike Hahne, Clarissa Müller, Suzan Yalman, Jessica Meißner, Manfred Kietzmann, Gerd Hamscher

**Affiliations:** 1 Institute of Food Chemistry and Food Biotechnology, Justus Liebig University Giessen, Heinrich-Buff-Ring 17, D-35392 Giessen, Germany; 2 Department of Pharmacology, Toxicology and Pharmacy, University of Veterinary Medicine Hannover, Foundation, Bünteweg 17, D-30559 Hannover, Germany

**Keywords:** antibiotics, amoxicillin, farm water, sulfadiazine, trimethoprim, HPLC-UV/DAD, stability

## Abstract

Due to the frequent use of veterinary drugs in animal husbandry, it is important to know their environmental behavior. In this context, little attention has been paid to the stability of the active ingredients in solutions prepared for administration. This is particularly problematic for antibiotics that trigger resistance when administered subtherapeutically. In order to investigate a possible influence of the preparation and storage of veterinary drugs on compound stability, three widely used antibiotics (amoxicillin, sulfadiazine, trimethoprim) were prepared in different model solutions. Depending on their individual stabilities, the incubation period lasted up to 70 days. Samples were analyzed at regular intervals by high-performance liquid chromatography–diode array detection and ultraviolet spectrophotometry. Following official recommendations, the investigations covered various parameters, e.g., pH, buffer substances, influence of light, and temperature. Sulfadiazine was incubated together with trimethoprim at concentrations of 120 mg L^−1^ and 80 mg L^−1^ for 70 days. Both compounds proved to be very stable under all experimental conditions and between 92 and 100% of the active ingredients remained. In 0.1% formic acid, a transformation product was found with less than 5% of the parent substance. In contrast, amoxicillin (500 mg L^−1^) was instable in almost all solutions under investigation. Within 17 days, the concentration of AMO decreased to 72% in ultrapure water. With the exception of a physiological saline solution, the amount of amoxicillin dropped below 10% or even below the detection limit. Thus, a physiological saline solution is best suited for the storage of dissolved amoxicillin for later administration.

## 1. Introduction

Pharmaceuticals are used in large quantities worldwide in both veterinary and human medicine [1,2,3]. The active ingredients make an indispensable contribution to a high level of health in humans and animals, as well as to the supply of animal foods. However, it is now very well-documented that many pharmaceuticals, due to their high persistence, can enter various environmental compartments after excretion by humans and animals [4].

Human and veterinary pharmaceuticals may only be used within various legal regulations and are subject to mandatory approval. In the case of veterinary medicinal products to be newly authorized, this also includes a comprehensive evaluation of the environmental properties of the substances and may result in restrictions on use [5,6]. In addition, the substances to be used in food-producing animals must be comprehensively tested with regard to their pharmacological–toxicological effects and their residue behavior, so that the safety of the food consumer is guaranteed.

Veterinary drug formulations are frequently used in intensive animal husbandry after appropriate dilution via drinking water [7,8,9]. In particular, in small animal practice, opened medications may be stored after dilution in the refrigerator or at room temperature for long periods of time. It is possible that the solvents used and the storage conditions influence the stability of the compounds and that there may be a loss of active ingredient. This is highly relevant, especially in the case of antibiotics, as subinhibitory concentrations may promote antibiotic resistance [10,11]. The spread of antibiotic resistance is seen as the greatest health and environmental risk of antibiotically active ingredients in both human and veterinary medicine [12,13]. As the problem is of global importance, a declaration of the G7 health ministers was adopted for the first time at the G7 meeting of the seven leading industrialized countries in Berlin in 2015 with the aim of making every effort to better protect people from multidrug-resistant germs and to prevent their spread [14].

The selection of substances for this study was based on the following considerations: Amoxicillin is globally one of the most important penicillins in both human and veterinary medicine. The instability of this class of compounds in the environment has been known for many years [15]. Sulfonamides are also frequently used together with trimethoprim, which acts synergistically. As an important representative of this class of compounds, sulfadiazine is used, e.g., in Germany, other European countries, and China. In general, sulfonamides show higher environmental stability than penicillins and can persist in manure, soil, and the aquatic environment [16,17].

All antibiotics were prepared in different model solutions and incubated for up to 70 days (sulfadiazine and trimethoprim) or 17 days, respectively (amoxicillin). Sampling was carried out regularly and quantitative analytical studies were performed by appropriate and partly validated high-performance liquid chromatography–diode array detection (HPLC-DAD) and ultraviolet spectrophotometry (HPLC-UV) methods. Considering ICH Q1A [18], the investigations were to cover relevant parameters (pH, buffer substances, influence of light, storage temperature and time, and influence of iron ions) including extreme conditions.

## 2. Results

### 2.1. Stability of Sulfadiazine (SDZ) and Trimethoprim (TMP) Solutions

**Figure 1:**
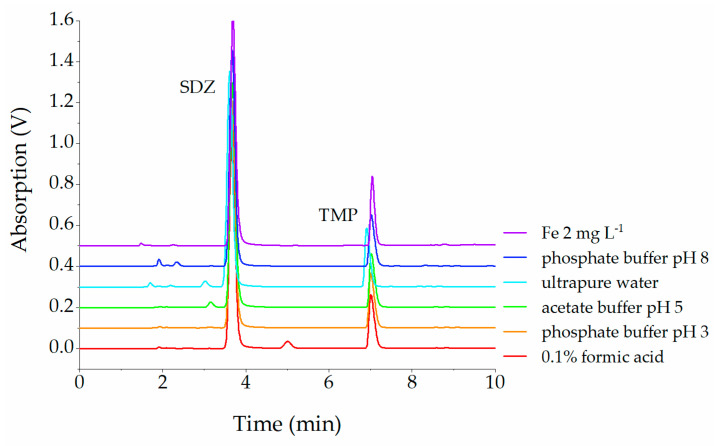
Stacked UV-chromatogram sections of various solutions of SDZ and TMP recorded at 270 nm. Samples were stored under the influence of light for 70 days at room temperature.

Over a period of 70 days, high stability was demonstrated for both sulfadiazine (SDZ) and trimethoprim (TMP). Figure 1 shows various UV chromatograms of SDZ and TMP after incubation in different aqueous solutions. Remarkably, the contents of SDZ and TMP remained between 92 and 100% in all solutions.

Consequently, only a few and predominantly very small peaks appeared in the chromatograms. In 0.1% formic acid, the largest peak eluted between SDZ and TMP at 5.5 min. The percent area relative to the sulfadiazine daily standard (120 µg mL^−1^) was less than 5% on day 70 under and without light influence.

### 2.2. Stability of Amoxicillin (AMO) Solution

**Figure 2:**
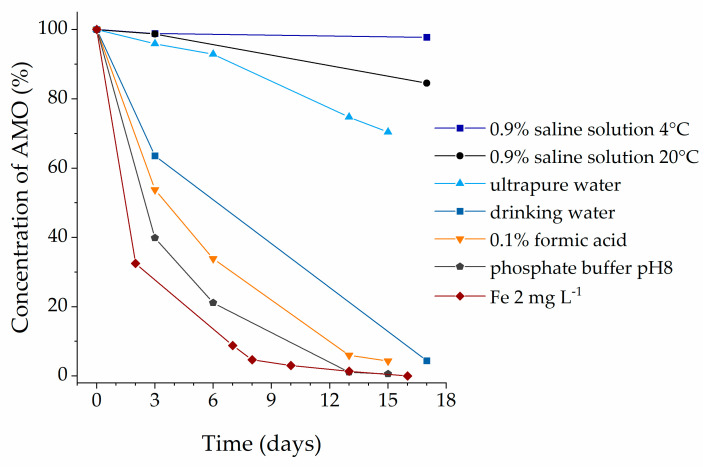
Elimination of AMO during illuminated storage of 17 days at room temperature (0.9% saline solution 4 °C stored at 4 °C in the dark).

Over a period of 17 days, high instability was demonstrated for AMO in most of the solutions. Please note that in a 0.9% saline solution no degradation occurred over the whole test period at 4 °C in the dark (see Figure 2).

**Figure 3:**
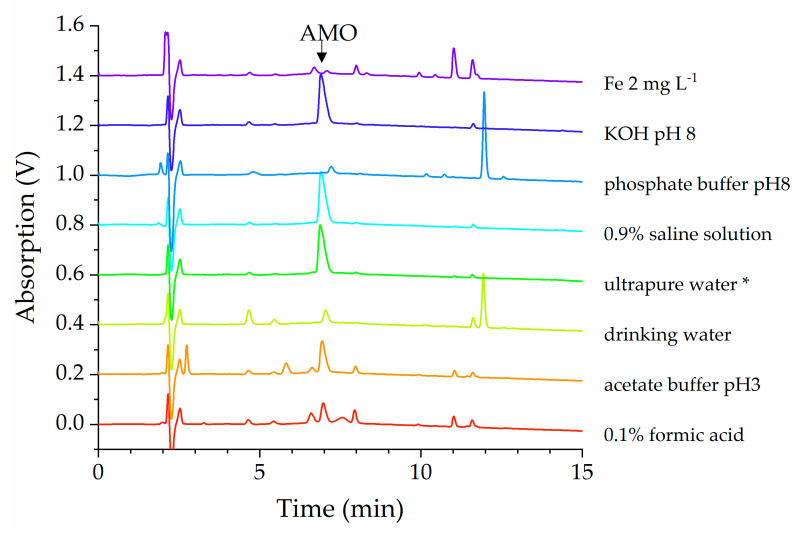
Stacked UV-chromatogram sections of AMO solutions detected at 230 nm. Samples were stored at room temperature for 10 days in the dark (* except ultrapure water, which was stored under light influence), AMO eluted at 6.7 min.

Despite the much shorter incubation period compared to SDZ/TMP, numerous new peaks appeared. For example, after 10 days without light influence, AMO degraded in all test solutions (see Figure 3). Many transformation products occurred within a broad physicochemical range.

According to chromatographic retention time, polar conversion products of AMO were formed in the acidic solution and the drinking water sample. In contrast, only very small peaks preceding AMO were detected in the other samples. Polar transformation products already known from the literature are amoxicilloic acid (AMO-A), the hydrolysis product of AMO, amoxilloic acid (AMA) formed by subsequent decarboxylation of AMO-A, and epimers [15,19,20]. Notably, in all solutions (except the physiological saline solution and ultrapure water) nonpolar transformation products of AMO occurred. In particular, a significant peak at 11.8 min was detectable in the alkaline sample with a phosphate buffer. Already identified nonpolar transformation products are amoxicillinpiperazine-2,5-dione and the stable 3-(4-hydroxyphenyl)-pyrazin-2-ol, which is discussed as a final degradation product [20].

## 3. Discussion

In intensive poultry and pig farming in particular, drug administration via drinking water is the method of choice [8,9]. This makes it possible to quickly supply even very large flocks with veterinary drugs. In this context, the question of how much active ingredient actually reaches the animal has rarely been asked. The same question arises as to what risks are involved in dissolving veterinary drugs in farm water. Since on-farm wells are often used, the quality of the water can vary greatly [21]. Important parameters in this context are, e.g., water hardness, pH, iron concentration, or microbiological conditions.

For this reason, the present study examined the extent to which the dissolution of active ingredients in defined solvents and their long-term storage influence the stability of the drugs. It is known from environmental studies that sulfonamides and trimethoprim commonly used in combination are quite stable [16,17,22,23]. ß-lactam antibiotics are considered to be markedly unstable under environmental conditions, especially at low pH, where they hydrolyze readily [15,19,24].

Distilled water, medium-hard drinking water, and a physiological saline solution were used as potentially well-suited solvents. To simulate strong acidic conditions, 0.1% formic acid was used, and for moderate acidic conditions an acetate buffer (pH 5) and for weakly basic conditions a phosphate buffer (pH 8) were applied. Since significantly higher iron contents are tolerated in farm water—in contrast to drinking water—an Fe^3+^ solution (2 mg L^−1^) was also used [21]. All solutions were incubated at 4 °C and 20 °C under and without exposure to light for up to 70 days.

The SDZ/TMP combination showed high stability in most solutions over the entire 70-day study period. Only under strongly acidic conditions, a transformation product with less than 5% of the parent substance was found in the solutions incubated at room temperature (Figure 1).

The behavior of amoxicillin, however, was quite different. Here, with the exception of the physiological saline solution and distilled water, less than 10% of the initial substance was found after 17 days. After 6 days, more than 50% of the initial substance had already disappeared (Figure 2). This was accompanied by the appearance of many new peaks in the chromatograms. From the elution behavior of the new compounds, it can be concluded that both more polar and less polar compounds were formed (Figure 3).

Thus, with a reasonable effort and a relatively simple HPLC analysis, the investigations carried out allow first conclusions to be drawn about the possible behavior of veterinary drugs administered via the drinking water.

The significance for veterinary practice can be estimated based on the fact that many veterinary drugs are administered via drinking water. For instable pharmaceuticals, such as amoxicillin, this may possibly lead to only a fraction of the active ingredient reaching the animal. In small animal practice, if necessary, only preparations dissolved with physiological saline solutions should be used, which can then also be stored in the refrigerator for a longer period. In large animal practice, medication via the animal feed is recommended in case of proven unfavorable behavior in the farm water.

## 4. Final Conclusions and Future Perspectives

The presented investigations certainly have a pilot character and should be extended, e.g., by including further critical solvent components. Very recently, Ecke et al. [20] described divalent copper and zinc ions as relevant to the degradation of AMO in the aquatic environment. Since on-farm wells are often used in large animal practice, the water composition depends on the soil and its individual composition. Therefore, a more comprehensive study with different water source locations should be conducted. The substance spectrum should be extended to quantitatively relevant veterinary drugs administered via the farm water. In this respect, substances from the tetracycline, fluoroquinolone, and macrolide classes should be considered. From the analytical, toxicological side, the elucidation of at least the main transformation products would be desirable. In addition, the characterization of their microbiological (residual) activity and possible toxic effects should be goals of future investigations.

## 5. Materials and Methods

### 5.1. Reagents and Materials

Analytical standards of amoxicillin (AMO), sulfadiazine (SDZ), and trimethoprim (TMP) were purchased from Sigma-Aldrich (Taufkirchen, Germany) in the VETRANAL grade.

HPLC grade methanol and acetonitrile were obtained from J. T. Baker (Avantor Performance Materials, Gliwice, Poland). Formic acid and ammonium acetate were acquired from Bernd Kraft (Duisburg, Germany) and Merck (Darmstadt, Germany), respectively. Iron solution and sodium acetate trihydrate were obtained from Carl Roth (Karlsruhe, Germany), and natrium chloride and potassium hydroxide were obtained from Merck KGaA (Darmstadt, Germany). The phosphate buffer consisted of KH_2_PO_4_, Na_2_HPO_4_∙2 H_2_O from Merck Millipore (Merck KGaA, Darmstadt, Germany) and ortho-phosphoric acid from Carl Roth (Karlsruhe, Germany).

Drinking water was obtained from Stadtwerke Giessen AG as a medium-hard water with a composition of cations natrium (5–9 mg L^−1^), potassium (1–3 mg L^−1^), magnesium (6–17 mg L^−1^), calcium (28–36 mg L^−1^), anions chloride (6–14 mg L^−1^), fluoride (<0.1 mg L^−1^), sulfate (8–36 mg L^−1^), hydrogen carbonate (109–153 mg L^−1^), and nitrate (12.8 mg L^−1^) (composition from Stadtwerke Giessen und Mittelhessische Wasserbetriebe, as of July of 2022).

### 5.2. Chemical Analysis

SDZ and TMP were determined with an HPLC UV system from Shimadzu (Kyoto, Japan). HPLC was performed using a low-pressure pump LC-10AD VP, autosampler SIL-10AD VP, degasser DGU-14A, oven CTO-10AC VP, and controller SCL-10A VP with SPD-10A VP as the UV detector combined with Class VP (Version 6.13 SP1, Shimadzu). Measurements were conducted on a 150 mm × 4.6 mm, 5 μm Hypersil GOLD column (Thermo Scientific, Dreieich, Germany) using gradient elution with 1 mM ammonium acetate in 0.5% formic acid (A) and methanol (B) with a total time of 14 min. The binary gradient started with an initial ratio of 15% of B. The ratio increased to 45% of B within 6 min and then to 80% of B in 1.5 min. This ratio was held constant for 1 min. Afterwards, the ratio was set back to the start condition with 15% of B within 1 min and held for 4.5 min for equilibration. The flow rate was set at 1.0 mL min^−1^, the injection volume at 10 µL, and the detection wave length at 270 nm with 30 °C for the oven.

To determine the limit of detection (LOD) and limit of quantification (LOQ), variance homogeneity had to be present for the corresponding calibration curve. To determine the variance homogeneity, a calibration curve was established from ten equidistant SDZ (0.05 to 0.5 μg mL^−1^) and TMP concentrations (0.005 to 0.05 µg mL^−1^). The calibration curve fulfilled the requirement of variance homogeneity in the investigated range. Thus, the LOD and LOQ could be calculated according to DIN 32645 by the calibration method. The LODs were 0.031 and 0.004 µg mL^−1^ for SDZ and TMP, and the calculated LOQs were 0.107 and 0.014 µg mL^−1^. External calibrations curves for the quantification of the samples were constructed between 50 and 150 µg mL^−1^ for SDZ (*y* = 91,074*x* − 256,107; *R*^2^ = 0.9993) and 50 and 150 µg mL^−1^ for TMP (*y* = 30,199*x* − 208,647; *R*^2^ = 0.9995).

In addition, an interday standard was added to each measurement, indicating the repeatability of the different measurement days. On each of these days, an intraday standard was also determined at the beginning and end of the measurement. For large sample sets, intraday standards were measured within the sequence. The determination of the deviations allowed us to check whether the quality of the HPLC analysis was guaranteed over the entire period. With 0.22%, a very good RSD was determined for both substances.

The determination of AMO was performed with an HPLC UV-DAD system from Shimadzu, Kyoto, Japan) with the same instrument of determination of SDZ and TMP combined with Class VP (Version 6.14 SP2A, Shimadzu). Measurements were conducted on a 150 mm × 4.6 mm, 5 μm Hypersil GOLD column (Thermo Scientific, Dreieich, Germany) using gradient elution with 0.1% formic acid in 2% acetonitrile (A) and acetonitrile (B) with a total time of 27 min. The binary gradient started with an initial ratio 3% of B and held this for 2.5 min. The ratio increased to 30% of B within 11.5 min and then to 80% of B in 5 min. This ratio was held for 1.5 min. Afterwards, the ratio was set back to the start condition with 3% of B within 1.5 min and held this for 5 min for equilibration. The flow rate was set at 1.0 mL min^−1^, the injection volume at 20 µL, and the detection wave length at 230 nm and 260 nm at 22 °C. By use of an external calibration curve from 0.5 to 120 µg mL^−1^, the AMO was quantified with a coefficient of determination R^2^ greater than 0.995 (*y* = 29,154*x* + 562; *R*^2^ = 0.9992). The signal-to-noise (S/N) ratio approach was used for the calculation of LOD and LOQ for AMO. Based on a S/N ratio of 3:1, the LOD of the method was 0.06 µg L^−1^. Applying a S/N ratio of 10:1, the LOQ was 0.18 µg L^−1^. With 0.85%, a good RSD was determined for AMO.

**Figure 4:**
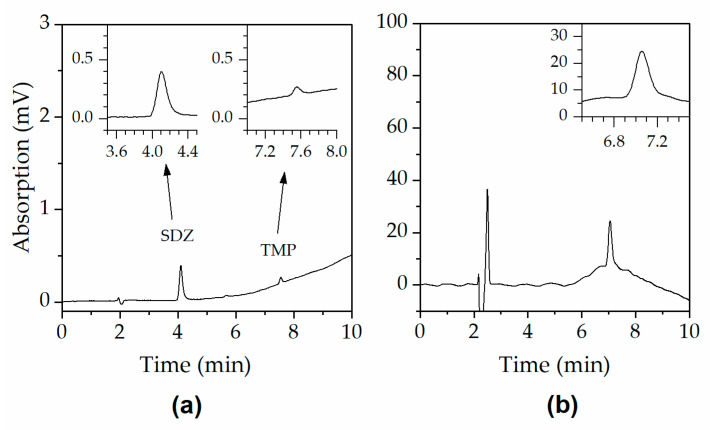
UV-chromatograms at the lowest calibration level of all compounds under investigation: (**a**) SDZ 0.05 μg mL^−1^, TMP 0.005 μg mL^−1^, and (**b**) AMO 0.5 μg mL^−1^.

Representative chromatograms at the lowest calibration level of all compounds under investigation are shown in Figure 4 (SDZ 0.05 μg mL^−1^, TMP 0.005 μg mL^−1^, and AMO 0.5 μg mL^−1^).

### 5.3. Sample Preparation

Stock solutions of antibiotics were prepared in methanol (SDZ 2 mg mL^−1^ and separately TMP 0.8 mg mL^−1^ and AMO 4 mg mL^−1^). These stock solutions were diluted in 50 mL tubes to target concentrations of SDZ (120 µg mL^−1^) with TMP (80 µg mL^−1^) and AMO (500 µg mL^−1^) in various solutions (0.1% formic acid, acetate buffer of pH 3 or 5, phosphate buffer of pH 3 or 8, ultrapure water and, especially for AMO saline solution (0.9% natrium chloride), drinking water, and iron solution with 2 mg L^−1^). The different solutions were stored at room temperature under and without light influence or in the fridge at 4 °C without light influence. Due to the individual stabilities of the active ingredients, an incubation time of 70 days was chosen for SDZ and TMP and 17 days for AMO. Prior to measurement, the samples treated with AMO were diluted 1 to 5 with high-purity water.

## Data Availability

The data presented in this study are available on request from the corresponding authors.
